# The Relationship between Psychosocial Factors and Cognition in Multiple Sclerosis

**DOI:** 10.1155/2017/6847070

**Published:** 2017-05-06

**Authors:** Fahad D. Alosaimi, Alaa AlMulhem, Mario Moscovici, Hanan AlShalan, Mohammad Alqazlan, Abdulgader Aldaif, Sanjeev Sockalingam

**Affiliations:** ^1^Department of Psychiatry, King Saud University, Riyadh, Saudi Arabia; ^2^College of Medicine, King Saud University, Riyadh, Saudi Arabia; ^3^Centre for Mental Health, University Health Network, Department of Psychiatry, University of Toronto, Toronto, Canada; ^4^Department of Mental Health, King Faisal Specialist Hospital and Research Centre, Riyadh, Saudi Arabia; ^5^Department of Neurology, King Saud University, Riyadh, Saudi Arabia

## Abstract

**Introduction:**

Multiple sclerosis (MS) is a common disorder in some regions of the world, with over 2.3 million people diagnosed worldwide. Cognitive impairment is one of the earliest symptoms to present in the course of the disease and can cause significant morbidity. We proposed a study to explore the psychosocial predictors of cognitive impairment in MS patients in Saudi Arabia, a previously unexplored patient population.

**Methods:**

Demographic data, depression scale (PHQ9), symptom burden (PHQ15), anxiety (GAD7), disease duration, and Montreal Cognitive Assessment (MOCA) scores were collected from 195 patients in a neurology clinic in Ryiadh, Saudi Arabia. Univariate and multiple regression analyses were conducted to identify variables that are significantly associated with cognitive impairment.

**Results:**

Variables that were identified to be significantly associated with cognition, *p* < 0.05, were education level, disease duration, and family history.

**Discussion:**

Both education level and disease duration were variables identified in previous studies. We showed family history to be a significant variable, and no association was found with depression or anxiety, which is unique to our study population.

**Conclusions:**

We identified several psychosocial predictors that are associated with cognition in our patient population. It was also noted that a difference exists between patient populations, highlighting the need for further studies in specific geographical regions.

## 1. Introduction

Multiple sclerosis (MS) is a chronic and recurrent neurological disorder with approximately 2.3 million people diagnosed worldwide, spanning every region around the world [1]. Multiple sclerosis causes significant morbidity [2] and mortality in young adults with a reduced average lifespan of less than 6 to 7 years [3]. Of particular significance is the high prevalence of cognitive symptoms in patients diagnosed with MS, which impacts overall quality of life [4]. Cognitive symptoms can be one of the earliest symptoms and are present in up to 70% of patients [5, 6]. In fact, cognitive impairments in particular as well as depression, fatigue, and motor function resulting from MS have been shown to contribute to lowered work performance [7] and increased unemployment rates [8–11], reduced social activities [12], long-term disability [10], mental health [13], and overall quality of life [14].

Cognitive impairment has been reported from the earliest stages of MS and is considered one of the main manifestations of the disease [15, 16]. Early stages of MS have been associated with significant cognitive impairment focused on attention, executive functions, memory, and learning [16]. Information processing in particular is the cognitive domain most widely affected by MS and is notably the first deficit to emerge [17–19].MS patients with progressive disease course, especially secondary progressive, can also experience a range of cognitive deficits and associated disability [20]. MS has been associated with delayed recall performance and lower acquisition of verbal memory [21] and episodic short-term memory [22]. In addition, MS can also result in slow processing speed [23], which has been linked to executive function deficits [24].

Studies have attempted to elucidate the relationship between psychosocial variables and cognition in patients with MS. Patients with MS exhibiting depressive symptoms show increased propensity to experience cognitive symptoms, thus contributing to disability and disease duration [13]. Other studies have suggested that mood is a strong predictor of cognitive function [12, 25]. Specifically, depression was found to affect executive function [26]. However, each study reported different predictors as being more important, either fatigue or low mood, emphasizing the need for further studies. Borghi et al. have examined multiple predictors, highlighting anxiety and depression as having impact on cognition [6]. The overall symptom burden or correlation to MS lesions was not examined in this study. Interestingly, self-reported cognitive impairment has also been showed to strongly correlate with fatigue and depression, both prominent complaints in MS [27]. In addition, there is a strong correlation between the presence of active lesions and cognitive decline when compared to dormant lesions [28]. Despite this literature, variability in results and the lack of studies looking at multiple predictors in the context of function and MS lesion activity highlight the need for further research.

Several studies have also explored the variation in different populations on MS outcomes, specifically the prevalence of cognitive impairment and degree of fatigue and depressive symptoms. A 10-year longitudinal study done by Chruzander et al. in Sweden showed that cognition, fatigue, and depression affect morbidity and mortality. The proportion of individuals with depression was found to be 18% which was associated with poor cognition and worsening disability [10]. A similar study performed in Italy showed a depression rate of 25%, which was not noted to be different than the general population [29]. Interestingly, both studies commented on variation in predictor prevalence, which they deemed surprising. Similar studies have been performed in Latin American [30] and Sicilian [31] patient populations with varying results. Interestingly, the authors discussed possible cultural differences and perception of cognitive symptoms in their study group as a possible reason for the differences in these subpopulations [32]. Nevertheless, there is limited literature available examining different patient populations and comparing predictors of cognition in MS. Examining different patient population can lead to a better understanding of the disease process and an improved ability to provide individualized treatment for better outcomes and improve outcomes in MS treatment [6].

This cross-sectional study aims to study psychosocial predictors of cognitive impairment in MS and provide enhanced understanding of differences in population-dependent outcomes. Specifically, we aim to focus on the impact of psychosocial predictors of cognitive impairment in Middle Eastern MS patient population, which serves as an understudied patient population. Specifically, the study looks at the impact of depression, anxiety, and pain on cognitive impairment in a Saudi Arabian patient population with MS.

## 2. Methods

### 2.1. Patient Recruitment

A total of 195 patients with MS were recruited from 203 consecutive patients seen in a neurology clinic at two hospitals in Ryiadh, Saudi Arabia: King Khalid University Hospital and King Faisal Hospital. A total of 158 female patients (81%) and 37 (19%) male patients were recruited between January 2014 and June 2015. All the patients in the study were 18 and over and were formally diagnosed with MS according to the McDonald criteria [33] with a neurologist confirming the diagnosis of MS. Patients included in the study group had the relapsing-remitting, primary, and secondary progressive MS. Patients had to be able to speak and read in Arabic language in order to complete study questionnaires. Patients with cognitive impairment due to other medical conditions were excluded from this study.

Informed consent was obtained during follow-up with a research assistant from patients that were interested in participating in the study. Ethics approval was obtained from the ethics review board at the Faculty of Medicine in Riyadh, Saudi Arabia.

### 2.2. Study Measures

Study participants underwent a series of assessments to collect demographic and clinical data. Demographic data, including age and sex, as well as information on the clinical features of MS (e.g., MS duration), were gathered by the study research assistant and clinic neurologist. Age was analyzed in the logistic regression in age groups of 10 years difference (18 to 30 years, 30 to 40 years, etc.). A visual analogue scale (VAS) for adherence was used to assess patient compliance with MS therapy as recommended by the neurological team [34].

Cognition was assessed using the Arabic version of the Montreal Cognitive Assessment (MOCA) to determine degree of cognitive impairment [35]. The MOCA has been used to assess the degree of cognitive impairment in participants. The MOCA was administered by a research assistant clinically trained to administer the assessment. The MOCA yields a total score with a maximum of 30, and cut-off scores for cognitive impairment have been defined as follows: A score of 26 and above is defined as normal, 25 to 23 as mild cognitive impairment, 23 to 11 is moderate cognitive impairment, and 10 and below is severe cognitive impairment [36]. The MOCA has good internal consistency with a Cronbach alpha of 0.83 and excellent positive (89%) and negative (100%) predictive values for mild Alzheimer's disease [37].

Depressive symptoms were assessed by administering the Arabic version of the PHQ9 questionnaire, which has been extensively used in MS patient population [38–40] to assess depressive symptoms. PHQ9 has also been used in the Arabic population for depression screening with similar cut-offs and thresholds [41]. The PHQ9 consists of 9 items scored from 0 to 3, and PHQ9 thresholds for mild, moderate, and severe depression are 5, 15, and 20, respectively. The PHQ-9 has been shown to have 88% specificity and 88% sensitivity for depression using a score of ≥10 when administered in primary care populations [42].

Similarly, a validated version of the Arabic version of the standardized GAD7 was used to assess anxiety symptoms and has been previously used in MS patient populations [43, 44]. Thresholds for anxiety severity on the GAD7 are 5 for mild, 10 for moderate, and 15 for severe. The GAD7 has good sensitivity (S) and specificity (Sp) for specific anxiety disorders: panic disorder (S = 0.74, Sp = 0.81), social anxiety disorder (S = 0.72. Sp = 0.82), generalized anxiety disorder (S = 0.89, Sp = 0.82), and posttraumatic stress disorder (S = 0.66, Sp = 0.91) [45]. The GAD7 has high internal consistency, with a Cronbach alpha = 0.79–0.91 [46].

PHQ-15 was used to measure the physical symptoms including fatigue and measures physical symptom burden for patients. The PHQ15 has a Cronbach alpha of 0.80, which suggests excellent internal validity [47]. The PHQ15 commonly uses a scale of 0 to 30 where 30 indicates higher symptom burden. PHQ-15 was not available in the Arabic language at the time the study was conducted, and an Arabic version of the PHQ15 was validated for the purpose of this study. PHQ-15 was first translated into Arabic by a linguistic specialist, fluent in both English and Arabic. Then, another specialist, fluent in both English and Arabic, carried out back translation into English. During this time, the back translation and the original scale were compared and any differences were discussed and resolved. Then, the scale was reviewed by content experts in psychosomatic medicine who were also fluent in both languages. The study questionnaire and all the five scales were then piloted on 20 individuals before the study began. The wording and suggested answers were modified based on the feedback from the pilot sample response.

### 2.3. Statistical Analysis

Mean and standard deviations were reported for continuous variables, and frequencies and percentages were reported from categorical variables. IBM SPSS was used to conduct a univariate linear regression and a multiple logistic regression analysis with the dependent variable being the MOCA score. The univariate analysis used each individual predictor versus the dependent variable to test variables in order to eliminate nonsignificant previously studied variables [48] from this patient population. We included nonsignificant demographic variables if there was a clinical rationale or preexisting research to demonstrate their relevance in the final model due to low statistical power. The MOCA score is defined in our study as a binomial variable with either none to mild cognitive or moderate to severe cognitive deficit (MOCA of 23 and below was considered moderate to severe cognitive deficits). Odds ratios and *p* values for each individual variable were reported with a *p* < 0.05 or lower for statistical significance.

## 3. Results

Demographic data for the 195 participants is summarized in Table 1. Most patients were female (*n* = 158, 81.0%), and 124 (63.9%) patients had completed a bachelor degree or more. Patients were evenly divided into categories of single or married, with 73 (50.8%) married. None of the patients in the study were common-law. Most patients were unemployed at the time of the study with 73 (37.4%) patients either having a full or part-time job. Patient mean age was 31.83 ± 8.94 years and had been diagnosed with MS for 6.33 ± 4.15 years. Mean MOCA scores for participants were 20.51 ± 4.67, with 4 patients (2.1%), 43 patients (22.2%), and 129 patients (66.5%) scoring in the severe, moderate, and mild cognitive impairment range, respectively. The average PHQ15, GAD7, and PHQ9 scores were 10.19 ± 5.51, 8.04 ± 5.83, and 9.28 ± 6.38, respectively.

### 3.1. Variables Associated with Cognitive Impairment

The result of the univariate logistic regression analysis is summarized in Table 2. Age and education at an elementary school level or lower were found to be significantly associated with cognitive impairment with *p* values of less than 0.05. On the other hand, family history of multiple sclerosis was significantly associated with lower cognitive impairment. Multiple logistic regression including all the variables in the model showed family history, education at elementary level or lower, and unemployment to be significantly associated with cognition seen in Table 3. The internal validity and reliability of our model were assessed using Cronbach coefficient and determined to be 0.68. Disease duration, family history, and an education at an elementary school level or lower were significantly associated with cognition.

## 4. Discussion

In this study, we identify several psychosocial factors that were associated with cognition in a unique patient population. Our study examined psychosocial predictors of cognition in the previously unexplored population of MS patients in Saudi Arabia. MS research into psychiatric comorbidities is limited in the Saudi Arabia population. Previously, Al-Deeb et al. have focused on epidemiological findings in the area [49]. Recently, a study has described rates of depression and anxiety in the Saudi population [50]. To date, an examination of specific psychosocial predictors and their relationship to cognition was not done in the Saudi population. We also included multiple variables that were previously explored to compare current literature findings, as well as unique variables, such as a patient's belief that MS is caused by “supernatural reasons,” and marital status. From our analysis, we discovered that two significant predictors that were negatively associated with cognition are unemployment and education level. Moreover, we also expect that higher education levels act as a cognitive reserve and buffer the potential impact on cognition. Both findings are consistent with the other studied patient populations and serve as psychosocial predictors [6, 10, 51]. It appears that family history of MS has a positive effect on cognition, a finding not previously described in specific patient populations to the best of our knowledge. It is possible that families more familiar with the diagnosis of MS may be more equipped to deal with symptoms of cognitive impairment and help their family members. Further work is necessary to explore this relationship.

We did not show any significant association between physical symptom burden (PHQ15), anxiety (GAD7), previous mental illness, sex, presence of other diseases, or employment status, which is consistent with previous findings. It has been shown that people that receive an MS diagnosis have difficulty in finding and maintaining employment as compared to the general population [8, 9], and our study shows association between unemployment and cognitive symptoms. Our average employment rate was 37.4% which is significantly lower than the national average around 90% [52] and is consistent with previous findings. However, it remains unclear if this is due to severity of cognitive symptoms, as previously postulated. Depression symptoms did not seem to influence cognitive impairment in our patient population, which challenges the findings of previous studies [6]. It is unclear why this association exists; however, it does highlight the need to further explore the potential for geographical differences between MS patients.

Limitations of our study include reliance on MOCA for cognitive assessment rather than more specific neurocognitive testing. Despite the fact that MOCA is a well-validated tool for assessing global cognition, it does not have the specificity of some neurocognitive tests that are more specific for MS. Using specific testing can highlight the particular cognitive domains that are affected and is a proposed future study. We did not show a significant association between disease duration and cognitive impairment, which could be related to our younger patient population or sample size. Studying a specific cultural population also limits the study generalizability; however, we demonstrate the possibility that cognition in heterogeneous patient populations can be better assessed by recognizing the impact of underlying cultural and geographical variables. Moreover, while physical symptom burden was not statistically significant as studied by the PHQ15 test, this questionnaire was not previously validated in Arabic-speaking patient populations and requires further validation. Finally, there have been previous studies that show worsening symptoms for patients with active versus nonactive MS lesions [15, 53]. It is possible that active MS lesions could serve as an interesting additional variable to include in future analyses of cognitive impairment in future studies.

## 5. Conclusions

In summary, we presented important psychosocial factors of cognition in MS patients, including disease duration, family history of MS, and education level. Differences in predictors from previous studies highlight the need for further validation in specific patient populations, which is particularly important for community practice. This study also highlights the need to monitor cognitive symptoms in patients where certain psychosocial factors are present.

## Figures and Tables

**Table 1:** Study participant characteristics.

Study characteristic	
Age (years)	31.83 ± 8.94
Sex (females)^∗^	158 (81.0%)
Education	
Bachelor degree or higher	124 (63.9%)
High school diploma	46 (23.7%)
Relationship status (married)^∗^	99 (50.8%)
Employment (employed)^∗^	73 (37.4%)
Duration of MS (years)	6.33 ± 4.15
PHQ15	10.19 ± 5.51
PHQ9	9.28 ± 6.38
GAD7	8.04 ± 5.83
MOCA	20.51 ± 4.67

^∗^Data listed as frequency (%); all other data listed as mean ± standard deviation. Note: Married included common-law; however, no participants were in a common-law relationship.

**Table 2:** Univariate logistic analysis examining relationship between variables and MOCA scores.

Variable	Odds ratio	Lower CI, upper CI	*p* value
Age group	1.05	1.01, 1.11	0.021^∗^
Sex (female versus male)	0.73	0.34, 1.58	0.425
Disease duration	1.02	0.95, 1.10	0.570
PHQ9	1.05	0.98, 1.11	0.370
GAD7	1.03	0.98, 1.08	0.283
PHQ15	1.02	0.96, 1.09	0.554
VAS	0.99	0.98, 1.01	0.229
Presence of other diseases	0.93	0.49, 1.79	0.811
Family history	0.31	0.15, 0.64	0.001^∗^
Previous mental illness	0.91	0.21, 3.92	0.898
Patient perception of MS etiology (attributing to magic or “evil eye” versus organic cause)	1.21	0.52, 2.48	0.611
Marital status (single or not)	0.823	0.52, 1.31	0.412
Education (bachelor or higher versus lower than bachelor degree)	1.55	0.61, 3.81	0.422
Education (elementary or lower versus bachelor degree)	6.38	1.40, 29.1	<0.001^∗^
Employment status (unemployed versus employed)	1.81	0.86, 2.11	0.052

^∗^Indicates significant variables where *p* < 0.05.

**Table 3:** Multiple logistic regression analysis examining relationship between variables and MOCA scores. Hosmer and Lemeshow (chi-square 9.96, *p* = 0.268).

Variable	Odds ratio	Lower CI, upper CI	*p* value
Age groups	1.04	0.99, 1.09	0.140
Sex (female versus male)	0.59	0.24, 1.43	0.245
Disease duration	1.03	0.94, 1.12	0.558
PHQ9	1.06	0.98, 1.14	0.137
GAD7	0.99	0.91, 1.07	0.799
Family history	0.292	0.13, 0.65	0.003^∗^
Education (bachelor or higher versus lower than bachelor degree)	1.95	0.84, 4.56	0.122
Education (elementary or lower versus bachelor degree)	5.522	2.06, 31.56	<0.001^∗^
Employment status (unemployed versus employed)	2.49	1.01, 3.61	0.011^∗^
Homemaker versus employed	1.08	0.32, 3.61	0.904
Student versus employed	0.371	0.11, 1.24	0.108

^∗^Indicates significant variables where *p* < 0.05.
